# Research on visual question answering based on dynamic memory network model of multiple attention mechanisms

**DOI:** 10.1038/s41598-022-21149-9

**Published:** 2022-10-06

**Authors:** Yalin Miao, Shuyun He, WenFang Cheng, Guodong Li, Meng Tong

**Affiliations:** Department of Information Science, Xi’an University of Technology, Xi’an, 710048 China

**Keywords:** Computational biology and bioinformatics, Computational neuroscience, Image processing

## Abstract

Since the existing visual question answering model lacks long-term memory modules for answering complex questions, it is easy to cause the loss of effective information. In order to further improve the accuracy of the visual question answering model, this paper applies the multiple attention mechanism combining channel attention and spatial attention to memory networks for the first time and proposes a dynamic memory network model (DMN-MA) based on the multiple attention mechanism. The model uses the multiple attention mechanism in the situational memory module to obtain the most relevant visual vectors for answering questions based on continuous memory updating, storage and iterative inference of the questions, and effectively uses contextual information for answer inference. The experimental results show that the accuracy of the model in this paper reaches 64.57% and 67.18% on the large-scale public datasets COCO-QA and VQA2.0, respectively.

## Introduction

The explosive growth of visual and textual data has caused more and more researchers to focus on cross-modal tasks that combine Computer Vision (CV) and Natural Language Processing (NLP), including cross-modal information retrieval^1^, image subtitles^2^, visual question answering^3^ (VQA), etc. The VQA model utilizes knowledge from both CV and NLP domains, where CV techniques are used to understand images and NLP techniques are used to understand questions, and both must be effectively combined to answer questions correctly.

After long-term text question answering research in the field of NLP, the answers to text question answering can be directly found in specific text descriptions or large knowledge bases. According to the correlation between visual question answering and text question answering, VQA extends visual information on the basis of text question answering. This has facilitated the progress of visual question answering research, but has undoubtedly brought more challenges. Images have higher dimensionality compared to textual information and more noise than plain textual information.In addition, natural language processing has tools such as parser and regular expression, while images lack language structure and grammar rules, so there is no tool for direct processing. Finally, the image captures richer information about the natural scene, and natural language represents a higher level of abstraction. For example, the phrase "a white skirt" does not fully describe the many possible patterns that the image can present.

In today's artificial intelligence developing, visual Q&A is regarded as a complete problem of AI, because it requires multimodal knowledge beyond a single domain, it makes the machine can process visual and linguistic information at the same time, which is important for improving human–computer interaction as part of the visual Turing test^4^, with a wide range of promising applications in scenarios such as early childhood education^5^ and medical treatment^6^^.^ In recent years, it has attracted the attention of researchers from multiple fields such as CV, NLP, and even knowledge graphs, and has become very popular throughout the academic field. A large number of data sets have been constructed and many models have been proposed.

**Figure 1:**
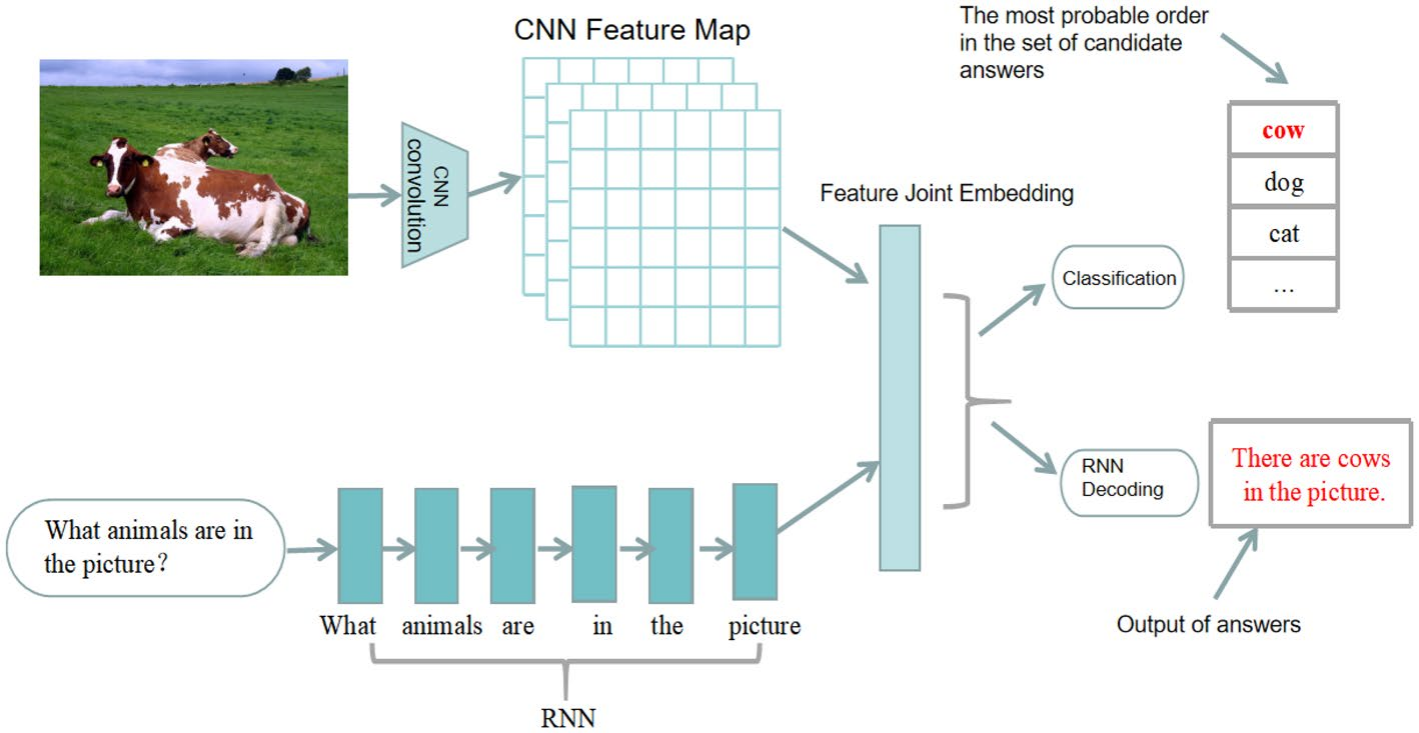
Overall framework of DMN-MA model.

The conceptual budding work of VQA^7^ restricts the defined question answers to 16 predefined base colors and 894 target categories. Mao et al.^3^ argued did not really define VQA and thus combined semantic segmentation of real-world scenes and symbolic reasoning about question statements in a Bayesian framework for automatic question and answer. Due to the excellent performance of Neural Networks in image classification, sequence translation and other tasks, Convolutional Neural Networks (CNN) and Recurrent Neural Networks (RNN) dominated the early models of VQA. Figure 1 below is the basic model of visual question-answering^8–11^.

In Fig. 1, the convolutional neural network is used to extract image features, and the recurrent neural network is used to characterize the semantic features of the problem, and then use multi-modal fusion methods such as splicing or element-wise multiplication to combine the multi-modal features from the picture and the problem Map to the same feature space to obtain the joint feature representation of the question and the picture to select the candidate answer with the highest probability from the predefined candidate answer set for output^9–11^; or input the joint feature representation to the LSTM recurrent neural network decoder to generate variable-length answers^8^.

## Related work

### Multiple attention

Previous VQA models used global image features to represent visual input^8–11^, which may provide irrelevant or noisy information for the answer prediction stage. Therefore, Yang et al.^12^ applied the attention mechanism to visual question answering for the first time based on the excellent performance of the attention mechanism in image subtitles. The model iteratively reasoned about the question and the image vector to gradually find the final target area.

Lu et al.^13^ believe that although attention mechanism has been introduced into VQA by some researchers, most of them only focus on the attention weight of the image area without considering the problem. Therefore, the author proposes two strategies of parallel and alternating collaborative attention to simultaneously focus on the image and problem. However, this model lacks the interaction between the two modes in order to avoid computational complexity.Since most traditional visual attention mechanisms for image captioning and VQA are top-down^12,13^, Anderson et al.^14^ proposed the BUTD model, which uses the object detection model to extract image features in visual question answering. Since the previous attention model independently calculates the attention distribution for each mode, ignoring the rich connection between vision and language, Kim et al.^15^ proposed the BAN model, which paid attention to the attention distribution of the two modes at the same time. Nguyen et al.^16^ proposed a dense two-way interactive attention model DCN to improve the accuracy of answer prediction. DCN is a completely symmetrical VQA model. Each question word corresponds to an image area, and each image area corresponds to a question word. Stack them to achieve multi-level interaction between images and questions. Domestic Yu et al.^17^ connected the two dense collaborative attention models of BAN and DCN in series to form the MCAN model and won the 2019 Visual Quiz Challenge.

Since the visual question answering attention mechanism in the existing visual question answering model generally only performs weighted pooling in the last convolutional layer of the image, where the receptive field is quite large, and the difference between the receptive fields is limited, resulting in insignificant spatial attention^18^. At the same time, different spatial regions have different weights, but different channels have the same weights, resulting in the unavoidable loss of feature map spatial information, which conflicts with the coexistence of spatial and channel characteristics of convolutional neural network feature maps. Therefore, researchers proposed to combine channel attention and spatial attention. This model was first applied to image captioning tasks by Chen et al.^19^. It is worth noting that image features are obtained by different filters in different channels and often have different semantic information. For example, some channels represent shapes and some channels represent colors.

**Figure 2:**
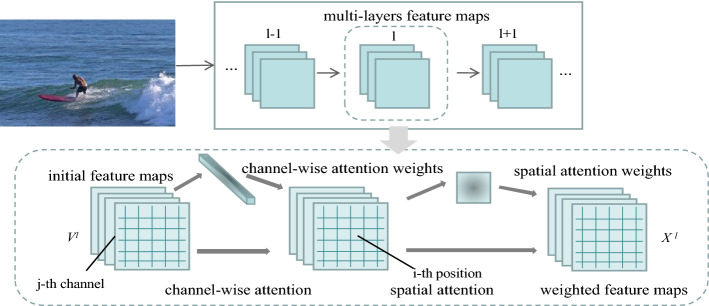
Overview of channel-wise and spatial attention.

Channel attention on the image feature map gives different weights to different feature maps, so channel attention is focused on objects, similar to "what". Spatial attention can be seen as attention to the "where" of the feature map, which focuses on location-related information. Channel attention and spatial attention work closely together as the "left and right arms" of the neural network, as shown in Fig. 2 below.

Because the channel attention mechanism can be regarded as the extraction of high-level semantics of images, and the high-level semantics enables visual information and textual information in visual question answering to perform answer inference in a common semantic space, reducing the semantic gap between multimodal features. In this paper, we first use question-guided channel attention on the last convolutional feature map of image features to select out the high-level semantics that are closely related to the question; next, we use a question-guided spatial attention mechanism on the attended channel feature map to select out the important spatial regions that answer the question, and this paper calls this method of combining channel attention with spatial attention as a multi-attention mechanism.

### Dynamic memory networks

Some of the questions in the visual Q&A involve the multi-hop relationship between objects, such as "What's in the bicycle basket?". The model needs to find the bicycle in the picture first, locate the position of the basket according to the bicycle, and then identify the objects contained in the basket. It can be seen that the visual Q&A answer prediction needs to gradually match the best picture area to answer the question according to the question. Attention provides an effective way to learn key information, but it lacks rich relational reasoning in the image. while it requires a lot of computing power to learn the attention weight distribution. In addition to using attentional mechanisms to extract the key information needed to answer questions, one should also have some memory capabilities to retrieve, reason and store relevant information according to different questions. Researchers have first tried to solve multi-step reasoning problems using modular architectures, with representative work on dynamic memory network^20^.

The dynamic memory network is a neural network model with a memory component and an attention mechanism. It is usually applied to question answering tasks, including text question answering and visual question answering. In visual question and answer, the dynamic memory network relies on the built-in attention mechanism of the network to iteratively update the memory vector to solve complex logical reasoning problems according to the problem. Memory network was first proposed by Weston et al.^21^ and applied to text question answering tasks. A certain memory mechanism is used for many neural network models such as RNN, LSTM and its variant GRU, but in the author's opinion, these memory modules are too small. However, the memory network is a model in the form of components. Each model is opposed to each other and affects each other. The network uses memory components to store scene information to achieve the function of long-term memory. The disadvantage is that this paper does not implement end-to-end training. Sukhbaatar et al.^22^ realized an end-to-end training method based on the literature ^21^ to repeatedly extract useful information, and realize multiple inferences in text question and answer. Kumar et al.^[23]^proposed Dynamic Memory Networks (Dynamic Memory Networks) based on memory networks and applied them to text question answering. The model includes four modules: input, question, episodic memory, and answer. In visual Q&A, Xiong et al.^20^ improved the initial DMN network input module and memory module, and verified the effectiveness of DMN in VQA. The DMN of Yan Ruyu et al.^24^ uses an object detection model in the image input module to extract image features and achieves a good accuracy rate on the visual question and answer data set.

In order to further improve the accuracy of the visual quiz model, this paper proposes a dynamic memory network model based on multiple attention mechanisms(Dynamic Memory Network with multiple Attention, DMN-MA), which mostly lacks the long-term memory module and cannot reason out the correct answer step by step according to the question. In the episodic memory module, this model captures effective contextual information for answering questions through multiple attention mechanisms based on question guidance, and performs multiple iterations and memory updates to achieve fine-grained questions and pictures Interactive.

## Method

### Network architecture

**Figure 3:**
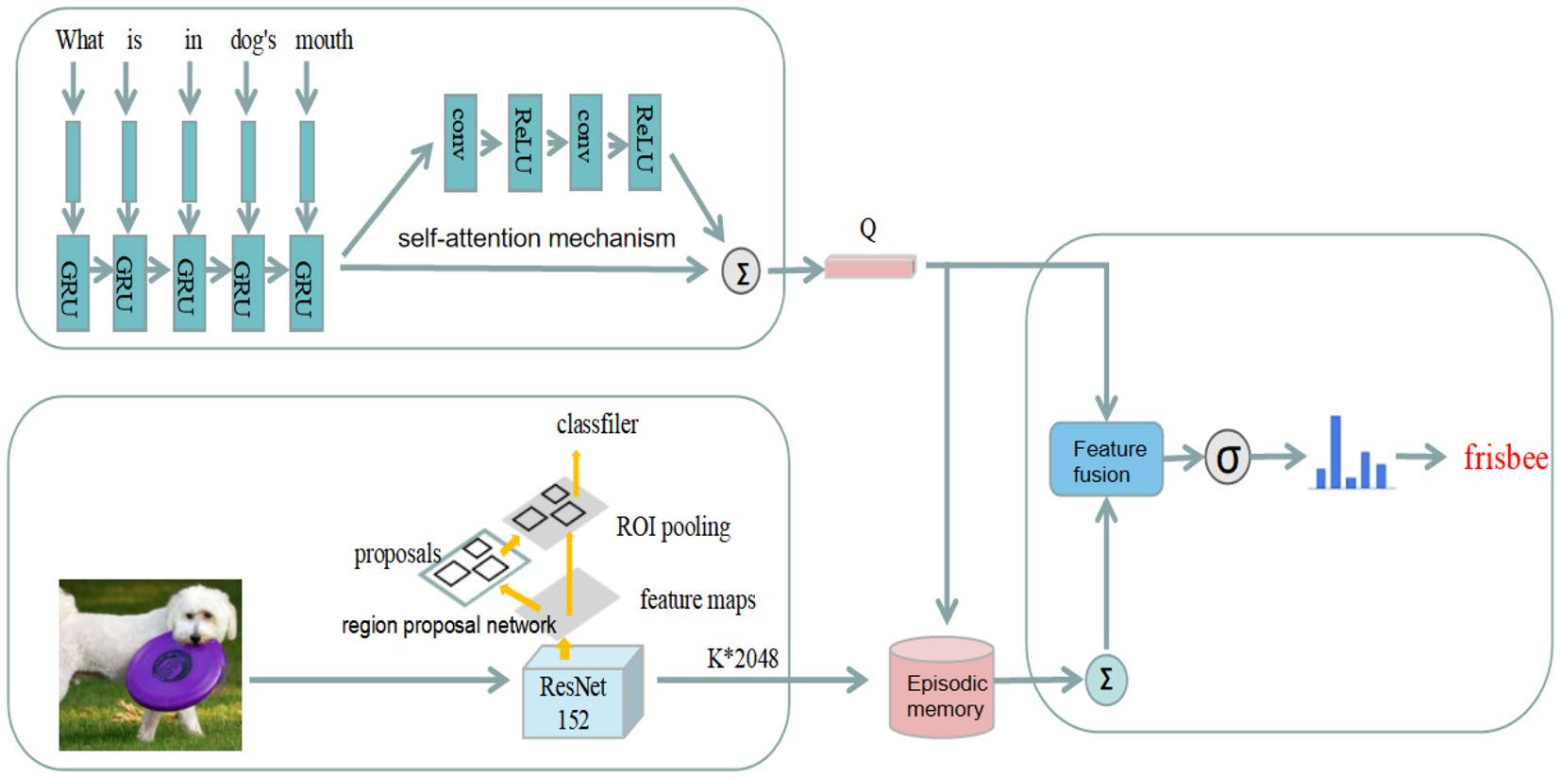
Overall framework of DMN-MA model.

The dynamic memory network model based on the multiple attention mechanism mainly consists of four parts: (1) Image input module, which is responsible for extracting image features. This article obtains target-level features, which scholars call the "bottom-up" attention mechanism. (2) Question input module, which extracts the features of the input question. This paper uses a self-attention mechanism on the question features. It is worth mentioning that the question text is preprocessed to a fixed length in this paper. (3) Episodic memory module. which uses multiple attention mechanisms to iteratively update the memory to generate the context vector needed to answer the question. (4) Multimodal fusion and answer prediction module, the module generates the answer based on the final memory vector and the problem vector. The model framework flow is shown in Fig. 3.

### Image input module

Because grid features often divide a complete object into multiple pieces, this chapter uses the pre-trained target detection model Faster R-CNN to extract image features, which is more in line with human visual attention. In this paper, the first K candidate regions with the highest confidence are selected as image features, and label each candidate box as D dimension. As shown in formula (1):

1 $$\mathrm{V}=\left[{\mathrm{v}}_{1},{\mathrm{v}}_{2},\dots ,{\mathrm{v}}_{\mathrm{K}}\right] , {\mathrm{v}}_{\mathrm{K}}\in {\mathbb{R}}^{\mathrm{D}}$$

### Question input module

Extract the problem feature vector, and represent the input problem as q $=[{q}_{1},{q}_{2},\dots ,{q}_{N}]$, where N is the sentence length. This paper uses the Glove^25^ word vector model pre-trained on a large corpus to obtain the word vector representation of each word, which is h $=[{h}_{1},{h}_{2},\dots ,{h}_{N}]$, where ${h}_{i}$ is the word vector of the word ${q}_{i}$. Input the word vector into the GRU network, and use the output of the last hidden layer of GRU as the sentence feature, as shown in the following formula (2):

2 $$S=ReLU\left(GRU\left({h}_{i}\right)\right), {h}_{i}\in {\mathbb{R}}^{P}$$

If the global feature of the question is directly used to predict the answer in visual question answering, the accuracy of the final answer may be affected. Therefore, this paper adds a text self-attention mechanism on the basis of sentence feature S, and obtains the final expression Q of the problem.

### Episodic memory module

**Figure 4:**
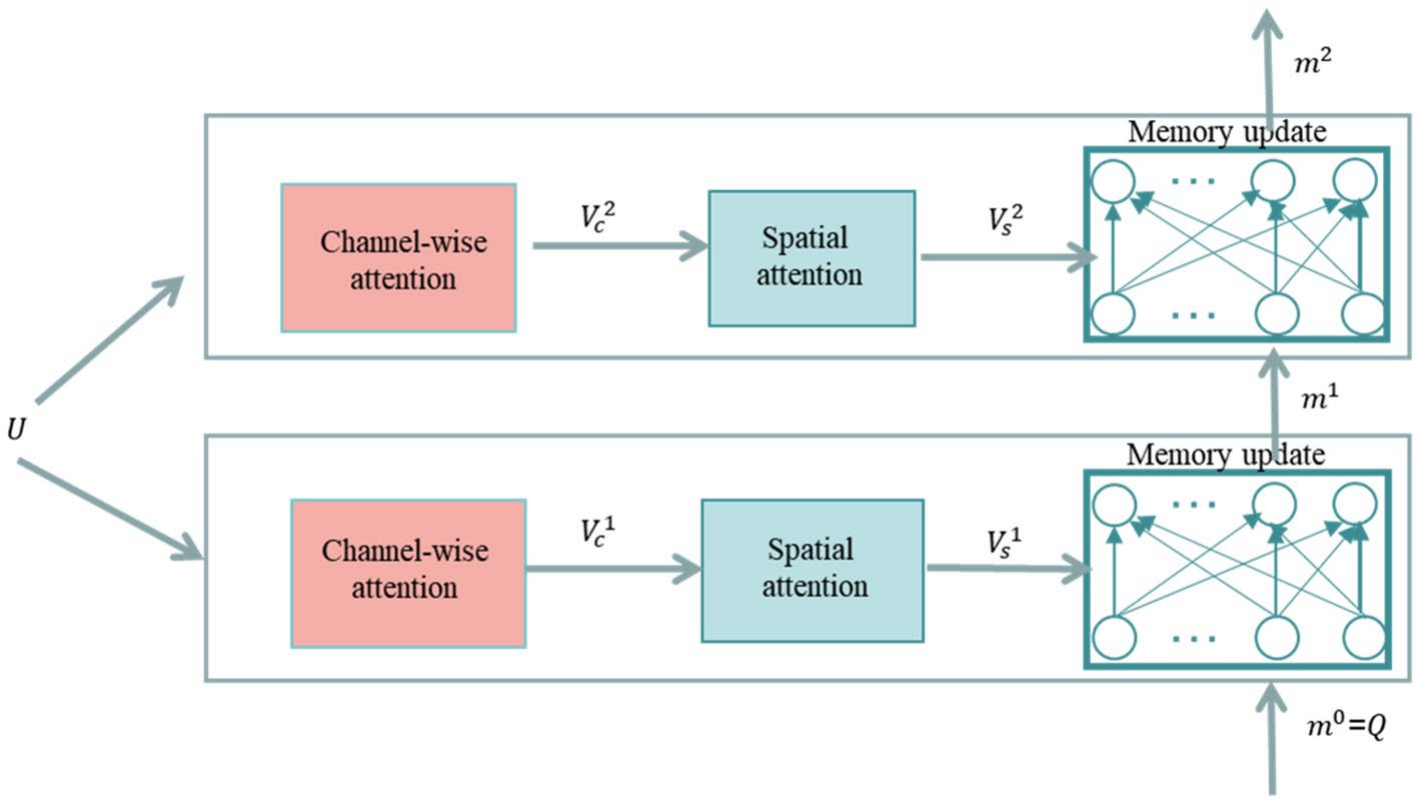
Schematic diagram of two iterations of episodic memory module.

In this paper, the episodic memory module of the dynamic memory network model uses multiple attention mechanisms to iteratively match the key visual areas in answering questions, which can be divided into three parts: channel attention, spatial attention and memory update. Figures 3 and 4 shows the flow chart of two iterations of episodic memory module.

As shown in Fig. 4, the visual features $\mathrm{V}=[{\mathrm{v}}_{1},{\mathrm{v}}_{2},\dots ,{\mathrm{v}}_{\mathrm{K}}]$ are first transformed into U,$\mathrm{U}=[{\mathrm{u}}_{1},{\mathrm{u}}_{2},\dots ,{\mathrm{u}}_{\mathrm{D}}]$, where ${\mathrm{u}}_{\mathrm{i}}\in {\mathbb{R}}^{\mathrm{k}}$, represents the i-th channel of the feature map and D is the total number of channels. Next, the mean pooling operation is used to obtain the feature maps for each channel, as shown in Eq. (3):

3 $$\mathrm{U}=\left[\overline{{\mathrm{u} }_{1}},\overline{{\mathrm{u} }_{2}},\dots ,\overline{{\mathrm{u} }_{\mathrm{D}}}\right]$$

where $\overline{{\mathrm{u} }_{\mathrm{i}}}$ is the mean pooling vector of ${\mathrm{u}}_{\mathrm{i}}$. For the first iteration, the channel attention module calculates the channel weights as shown in formulas (4) and (5):

4 $${\mathrm{b}}^{\mathrm{t}}=\mathrm{tanh}(({\mathrm{W}}_{\mathrm{vc}}^{\mathrm{t}}\overline{\mathrm{u} }+{\mathrm{b}}_{\mathrm{vc}}^{\mathrm{t}})\otimes ({\mathrm{W}}_{\mathrm{mc}}^{\mathrm{t}}{\mathrm{m}}^{\mathrm{t}-1}+{\mathrm{b}}_{\mathrm{mc}}^{\mathrm{t}}))$$

5 $${\upbeta }^{\mathrm{t}}=\mathrm{Softmax}({\mathrm{W}}_{\mathrm{c}}^{\mathrm{t}}{\mathrm{b}}^{\mathrm{t}}+{\mathrm{b}}_{\mathrm{c}}^{\mathrm{t}})$$

where ${\mathrm{W}}_{\mathrm{vc}}^{\mathrm{t}}$, ${\mathrm{W}}_{\mathrm{mc}}^{\mathrm{t}}$, ${\mathrm{W}}_{\mathrm{c}}^{\mathrm{t}}$ are embedding matrices,${\mathrm{b}}_{\mathrm{vc}}^{\mathrm{t}}$, ${\mathrm{b}}_{\mathrm{mc}}^{\mathrm{t}}$, ${\mathrm{b}}_{\mathrm{c}}^{\mathrm{t}}$ are bias terms, and $\otimes$ is outer product of vectors. Through the channel attention module, we get the channel attention vector, which is ${\upbeta }^{\mathrm{t}}$, ${\mathrm{m}}^{0}\mathrm{ is Q}$.

After obtaining the channel attention weight ${\upbeta }^{\mathrm{t}}$, it is fed back to the function ${\mathrm{f}}_{\mathrm{c}}$ to calculate the mapping graph ${\mathrm{V}}_{\mathrm{c}}^{\mathrm{t}}$ after channel attention update, as shown in formula (6):

6 $${\mathrm{V}}_{\mathrm{c}}^{\mathrm{t}}={\mathrm{f}}_{\mathrm{c}}({\upbeta }^{\mathrm{t}},\mathrm{V})$$

The ${\mathrm{f}}_{\mathrm{c}}$ function is the product of the channel map and the corresponding channel weight. The updated map is shown in formula (7)

7 $${\mathrm{V}}_{\mathrm{c}}^{\mathrm{t}}=\left[{\mathrm{v}}_{\mathrm{c}1}^{\mathrm{t}},{\mathrm{v}}_{\mathrm{c}2}^{\mathrm{t}},\dots ,{\mathrm{v}}_{\mathrm{ck}}^{\mathrm{t}}\right]$$

where ${\mathrm{v}}_{\mathrm{ci}}^{\mathrm{t}}$ represents the visual feature of the $\mathrm{i}$ object at the t-th iteration. Next, the spatial attention weight ${\upeta }^{\mathrm{t}}$ is calculated on the basis of channel attention, as shown in formulas (8 and 9).

8 $${\mathrm{a}}^{\mathrm{t}}=\mathrm{tanh}(({\mathrm{W}}_{\mathrm{vs}}^{\mathrm{t}}{\mathrm{V}}_{\mathrm{c}}^{\mathrm{t}}+{\mathrm{b}}_{\mathrm{vs}}^{\mathrm{t}})\oplus ({\mathrm{W}}_{\mathrm{qs}}^{\mathrm{t}}\mathrm{Q}+{\mathrm{b}}_{\mathrm{qs}}^{\mathrm{t}}))$$

9 $${\upeta }^{\mathrm{t}}=\mathrm{Softmax}({\mathrm{W}}_{\mathrm{s}}^{\mathrm{t}}{\mathrm{a}}^{\mathrm{t}}+{\mathrm{b}}_{\mathrm{s}}^{\mathrm{t}})$$

${\mathrm{W}}_{\mathrm{vs}}^{\mathrm{t}},{\mathrm{W}}_{\mathrm{qs}}^{\mathrm{t}}$ and ${\mathrm{W}}_{\mathrm{s}}^{\mathrm{t}}$ are the weight matrix of the t-th update, and ${\mathrm{b}}_{\mathrm{vs}}^{\mathrm{t}},{\mathrm{b}}_{\mathrm{qs}}^{\mathrm{t}}$ and ${\mathrm{b}}_{\mathrm{s}}^{\mathrm{t}}$ are the bias. It can be seen that these parameters are not shared in the iterative process. $\oplus$ represents the addition of matrix and vector. ${\upeta }^{\mathrm{t}}\in {\mathbb{R}}^{\mathrm{k}}$ represents the importance of each object area. In this paper, the updated feature map is obtained after the first channel attention, and then the multiple attention of spatial attention. The calculation method is shown in formula (10)

10 $${\mathrm{V}}_{\mathrm{s}}^{\mathrm{t}}={\mathrm{f}}_{\mathrm{s}}\left({\upeta }^{\mathrm{t}},{\mathrm{V}}_{\mathrm{c}}^{\mathrm{t}}\right)$$

Function ${\mathrm{f}}_{\mathrm{s}}$ is the product operation of spatial attention ${\upeta }^{\mathrm{t}}$ and the corresponding image feature ${\mathrm{V}}_{\mathrm{c}}^{\mathrm{t}}$.

In each passing of channel attention module and spatial attention module, this paper hopes to update episodic memory ${\mathrm{m}}^{\mathrm{t}-1}$ with new image feature ${\mathrm{V}}_{\mathrm{s}}^{\mathrm{t}}$ and generate ${\mathrm{m}}^{\mathrm{t}-1}$ vector. Following the work of Xiong et al., this paper uses ReLU activation function to update memory, and a new calculation method of episodic memory is shown in Formula (11):

11 $${\mathrm{m}}^{\mathrm{t}}=\mathrm{ReLU}({\mathrm{W}}^{\mathrm{t}}[{\mathrm{m}}^{\mathrm{t}-1};{\mathrm{V}}_{\mathrm{s}}^{\mathrm{t}};\mathrm{Q}]+\mathrm{b})$$

$[ \cdot ; \cdot ]$ represents feature splicing, ${\mathrm{W}}^{\mathrm{t}}$ is the matrix of parameter update, and $\mathrm{b}$ is bias.

### Multimodal fusion and answer prediction

The feature fusion module extracts complex and high-level interactions between question text semantics and image visual concepts, which plays a key role in the performance of VQA model.The final memory ${\mathrm{m}}^{\mathrm{t}}$ and problem vector $\mathrm{Q}$ are fused in the way of BLOCK multimodal fusion, which is the final fused feature $\mathrm{J}$.BLOCK ^26^ is one of the excellent methods of visual question answering and multimodal fusion, which greatly reduces the amount of model parameters.In this chapter, answer prediction is regarded as a multi classification problem.The DMN-MA model uses the $Sigmoid$ function to perform answer prediction, which allows multiple correct answers to each question, and each candidate answer has a score in the range (0, 1). In this paper, the candidate answer with the largest probability value is selected as the final answer of the model, as shown in Formula (12) below:

12 $$\mathrm{y}=\mathrm{Sigmoid}({\mathrm{W}}_{\mathrm{j}}{\mathrm{J}}^{\mathrm{^{\prime}}}+{\mathrm{b}}_{\mathrm{j}})$$

${\mathrm{W}}_{\mathrm{j}}$ is the parameter of the fully connected layer, ${\mathrm{J}}^{\mathrm{^{\prime}}}$ is the fusion vector $\mathrm{J}$ through max pooling, and ${\mathrm{b}}_{\mathrm{j}}$ is the bias term. The cross-entropy cost function is used in the training process.

### Statement

All authors of this article participated the study at the same time, and the model was proposed by Wenfang Cheng.

## Experiments

### Datasets

(1) COCO-QA data set

The COCO-QA^9^ data set is a representative attempt by researchers to improve the scale of the visual Q & A data set. The data set image is from the MS-COCO.A total of 123,587 images are included, among which 72,783 are used for training and 38,948 are used for testing. According to the answer types, COCO-QA data set questions are divided into 4 categories, namely Object, Number, Color and Location. In addition, the data set has a one-word answer for each question.

(2) VQA2.0 data set

The VQA2.0 data set^27^ contains 204,721 images from MS-Coco, and there are about 123,287 images in the training and validation sets, including 80,000 from the training set and 81,434 from the test set. Each picture has three questions, and each question has ten answers. The answers to each question are provided by ten different markers. To address the linguistic bias problem of the visual question–answer dataset, the VQA2.0 dataset associates each question with a pair of similar pictures, but the corresponding answers are not identical. The types of questions can be divided into three types: Yes /No, Number and Other. VQA2.0 is one of the widely used data sets in visual question answering.

### Metrics

In this paper, the proposed model is evaluated using the official evaluation metric^4^, i.e., the model predicts answers that are consistent with at least three annotators' provided answers in order to be considered as correct model predictions, as shown in Eq. (13) below:

13 $$\mathrm{Ans}=\mathrm{min}\{\frac{\#\text{number of ans human voted }}{3},1\}$$

### Implementation details

This paper uses Python3.6 and Pytorch 1.1.0 framework. Specifically, the image input module K = 100, and the feature vector dimension of each object is 2048. Resnet152 is used as the basic network for image feature extraction. All activation functions in the experiment used ReLU, and use a dropout of p = 0.5 in the input and output layers to prevent overfitting.During the training process, all training samples were randomly shuffled, the batch size was set as 32, and the epoch was 20.The ADAM stochastic gradient descent algorithm was used in the training process, and the initial learning rate was 0.001. After training 5 epochs, the DMN-MA model reduced the learning rate to 1/10 of the previous one after every 3 epochs.

### Experimental results

Due to the uncertainty of iteration times of DMN-MA model episodic memory module, this paper first set different iteration times in Coco-QA data set and VQA2.0 data set to find the best performance of the model. The experimental results of the overall accuracy and iteration times of the model in the two data sets are shown in Table 1.

**Table 1:** Comparison of iteration accuracy of episodic memory module.

Dataset	1 Time (%)	2 Times (%)	3 Times (%)	4 Times (%)	5 Times (%)
COCO-QA	63.68	64.31	64.57	63.77	61.58
VQA2.0	65.49	66.53	67.18	66.24	63.65

As can be seen from Table 1, when the number of iterations is 3, the overall accuracy of the model in the two data sets is the highest. Therefore, the number of iterations is set as 3 in the following experiment. In addition, the accuracy is the highest when the Number of iterations is 2 for both the Number problems of the two data sets. The author speculated that this might be due to the soft attention mechanism used in this paper. When the number of iterations increased, the model repeated the modeling candidate box, resulting in the objects in the image could not be correctly distinguished. In order to verify the validity of the model proposed in this chapter, Table 2 lists the experimental results of this chapter model and other mainstream methods on the COCO-QA test set.

**Table 2:** Accuracy compared to other methods in COCO-QA dataset.

Model	Overall (%)	Object (%)	Number (%)	Color (%)	Location (%)
GUESS^9^	6.65	2.11	35.84	13.87	8.93
VIS + LSTM^9^	53.31	56.53	46.10	45.87	45.52
VIS + BOW^9^	55.92	58.66	44.10	51.96	49.39
2-VIS + LSTM^9^	55.09	58.17	44.79	49.53	47.34
SAN^12^	61.60	64.50	48.60	57.90	54.00
QRU^9^	62.50	65.06	46.90	60.50	56.99
DMN-MA	64.57	66.82	49.56	61.73	58.48

GUESS means that the corresponding answer is randomly selected from the training set according to the type of question (the answer is usually "cat", "tow", "white", "room"). The VIS + BOW model sums the image features and the word vectors of the question, and performs polynomial logistic regression on the resulting vector to predict the answer. The VIS + LSTM model uses an LSTM to encode images and questions. The 2-VIS + BLSTM model inputs image features twice at the beginning and end of question encoding. The SAN model is one of the classic attention mechanism models for visual question answering tasks. This model uses the image attention mechanism to iterate multiple times to find the key image regions to answer the question. The QRU model iterates over each image region according to the question and selects the image region most relevant to the question to update the question representation, further giving the correct answer.

As can be seen from Table 2, the overall accuracy of DMN-MA model proposed in this chapter reaches 64.57%, compared with the traditional VIS + LSTM method, increased accuracy by 11.26%. In particular, compared with the classical attention method SAN, the overall accuracy is increased by about 3%, and compared with the QPU model, the accuracy is increased by 2.07%. In addition, it can be found that the model proposed in this chapter also performs well on all types of problems. Compared with the SAN model, the "Object" category increased by 2.32% and the "Location" category increased by 4.48%. Compared with the QRU model, the categories of "Number" and "Location" are significantly improved, which are 2.66% and 1.49% respectively. One likely reason is that this paper adopts the object detection model Faster-R CNN, which can locate and classify objects simultaneously, to extract image features, while other models adopt grid features. Therefore, other models are easy to lose the object space information in the process of multiple feature interactions. In addition, DMN-MA model uses self-attention mechanism in the problem semantics, which is very helpful to improve the model performance. This indicates that it is not enough to use spatial attention only for iterative reasoning in visual question-answering research, and that problem-based channel attention is equally important. In addition, in order to verify the generalization of the model, this chapter also compares the VQA2.0 standard test set with other advanced methods, and the results are shown in Table 3.

**Table 3:** Results of DMN-MA model in VQA2.0 test-standard split.

Model	Overall (%)	Yes/no (%)	Number (%)	Other (%)
Prior^28^	25.98	61.2	0.36	1.17
CNN + LSTM^29^	54.22	73.46	35.18	41.83
MCB^30^	62.27	78.82	38.28	53.36
ReasonNet^31^	64.64	78.86	41.98	57.39
BUTD^14^	65.67	82.20	43.90	56.26
MUTAN^32^	66.38	83.06	44.28	56.91
DMN-MA	67.18	84.23	45.03	57.76

Where Prior means that the model predicts the most common answer in the training set based on the question. CNN + LSTM uses CNN to extract image features for the model, uses LSTM network to extract question features, and finally uses point multiplication to perform multi-modal feature fusion. This model is often used as a benchmark model for VQA. ReasonNet models learn multimodal representations of images and questions through a modular neural network.

As shown in Table 3, the DMN-MA model proposed in this paper has an overall performance 12.96% higher than the benchmark model CNN + LSTM, 4.91% higher than MCB model, and 2.54% higher than RESONNET model. In addition, the overall accuracy of the model in this chapter is 1.51% higher than that of the classical BUTD model. It is worth noting that DMN-MA model and BUTD model adopt the same data preprocessing method, that is, Fast-RCNN is used to extract the visual features of the image, and Glove + GRU is used to extract the features of the problem. The difference is that BUTD model only uses the spatial attention mechanism for answer prediction. This fully proves the validity of the model proposed in this chapter.

### Visualization

**Figure 5:**
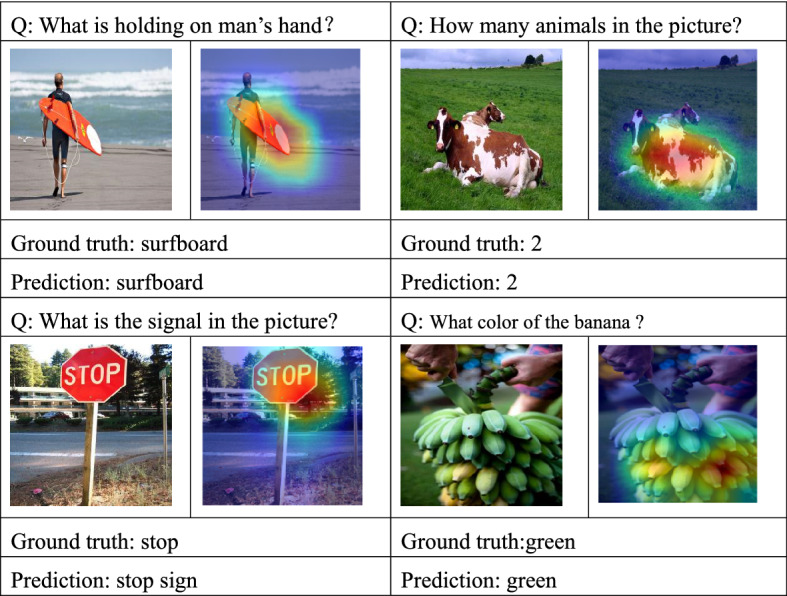
Visualization result.

Aiming at the model proposed in this paper, this chapter randomly selects several pictures and questions from the data set for visual display of attention, as shown in Fig. 5 below. The top of the picture is the question, the left picture is the original picture, the right picture is the picture after the model attention visualization, the bottom Ground truth is the answer of the data set, Prediction means the answer of the model.

Figure 5 shows the results of attention visualization of DMN-MA model. In this paper, the thermodynamic diagram is used to represent the attention weight of the image area. It can be seen that the model accurately locates the important area of the image and can correctly answer relevant questions, which further proves the effectiveness of the model proposed in this chapter.

## Conclusion

Unlike previous attention models, this paper does not only use the spatial-based attention mechanism, but further uses the channel attention mechanism, which makes the visual Q&A model use different weights on different channel feature maps, and the spatial attention mechanism becomes an effective complement to the channel attention mechanism. In addition, the input module and the situational memory module of the dynamic memory network model are studied in depth in this paper. In the input module, Faster-RCNN is used to obtain object features at the target level; in the situational memory module, multiple attention mechanisms are used to continuously update and store memories according to the questions, and iterative reasoning is performed to obtain the most relevant visual vectors to answer the questions, and contextual information is effectively used for answer reasoning. Finally, this paper fuses the final memory of the network and the question representation to infer the correct answer. It is validated on two publicly available datasets, COCO-QA and VQA2.0, for comparison with existing mainstream methods. The experimental results show that the DMN-MA model proposed in this chapter achieves better results in both the overall accuracy and various types of questions.

## Data Availability

Our research does not involve the study of human embryos, gametes and stem cells. The dataset in this paper comes from the VQA public dataset, and all the images used belong to this dataset. Here is the dataset article and download link: Goyal et al. ^27^. https://visualqa.org/. The datasets generated and/or analysed during the current study are not publicly available due the research is still in the process of experiment, some data are not suitable for open source, but are available from the corresponding author on reasonable request. Due to the raw/processed data required to reproduce these findings cannot be shared at this time as the data also forms part of an ongoing study.
